# Supplementary material for: Low Plasma Choline, High Trimethylamine Oxide, and Altered Phosphatidylcholine Subspecies Are Prevalent in Cystic Fibrosis Patients with Pancreatic Insufficiency
Source: Nutrients. 2025 Feb 28;17(5):868. doi: 10.3390/nu17050868 (PMC11901616; doi:10.3390/nu17050868)
Supplement: Supplementary file 1 [file nutrients-17-00868-s001.zip › nutrients-3486294-supplementary.pdf]

Supplementary file:

Low plasma choline, high trimethylamine oxide and altered phosphatidylcholine subspecies are prevalent in cystic fibrosis patients with pancreatic insufficiency

Wolfgang Bernhard[1], Anna Shunova[1], Julia Boriga[2], Ute Graepler-Mainka[2] and Johannes Hilberath[2]

Departments of [1]Neonatology and [2]General Pediatrics, Hematology & Oncology, Faculty of Medicine, Eberhard-Karls-University, Tübingen, Germany.

Corresponding address:

Wolfgang Bernhard, Dr. med., Dr. rer. physiol.

Department of Neonatology, Children's Hospital,

Eberherd-Karls-University, Calwer Straße 7, D-72076 Tübingen, FRG

Phone: #-49 174 3015025

E-mail: wolfgang.bernhard@med.uni-tuebingen.de

Supplementary Results

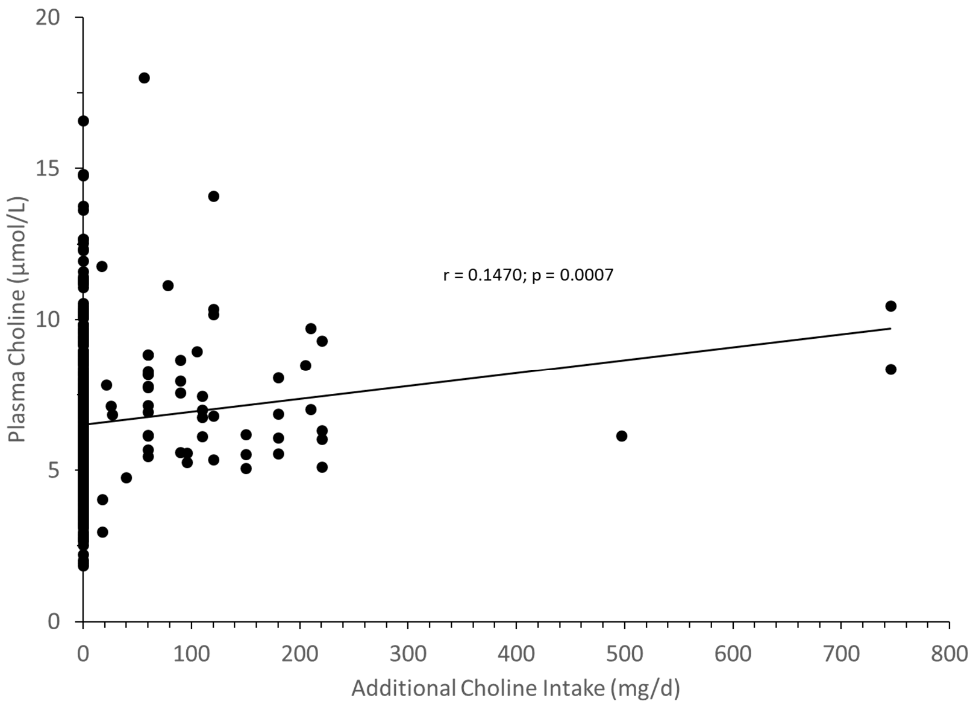

Figure S1: Plasma concentrations of choline in exocrine pancreas-insufficient (EPI) CF patients in relation to the intake of choline via food supplements (0-745mg/d). Data are from individual determinations of patient visitations. Abbreviations: ρ, Spearman correlation coefficient; p, significance level.

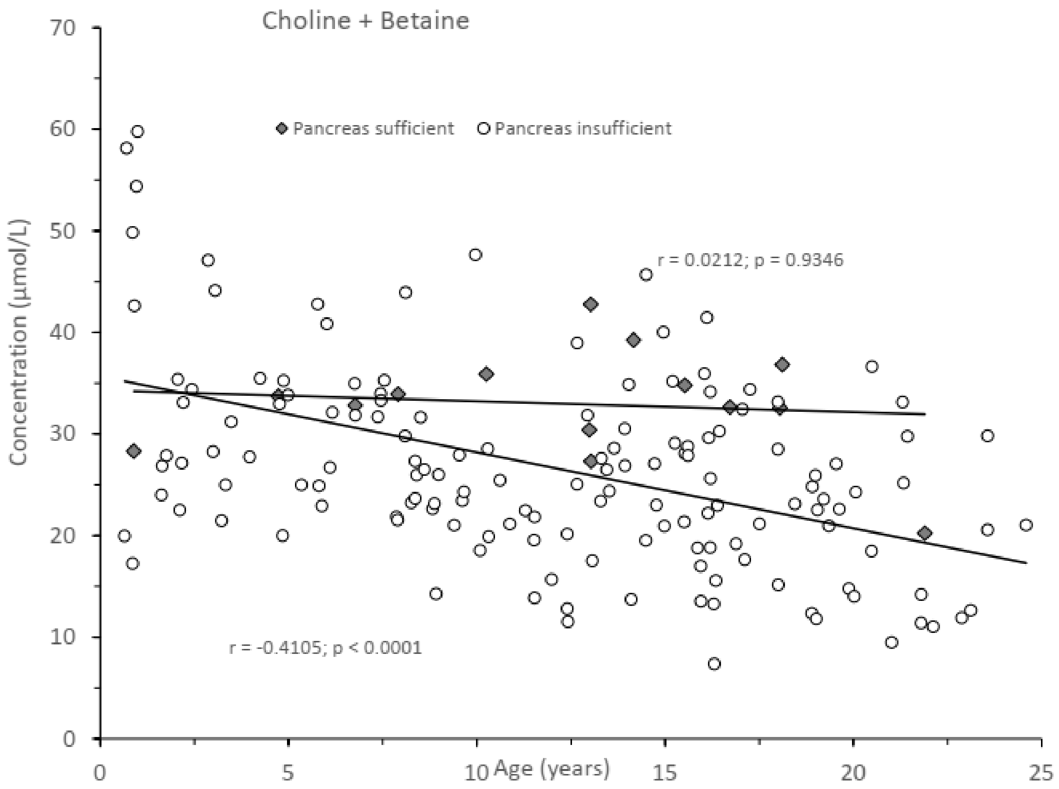

Figure S2: Plasma concentrations of the sum of choline + betaine in relation to the age of CF patients. Data are median values of individual CF patients with (N=148) and without (N=14) exocrine pancreas insufficiency. Abbreviations: ρ, Spearman correlation coefficient; p, significance level.

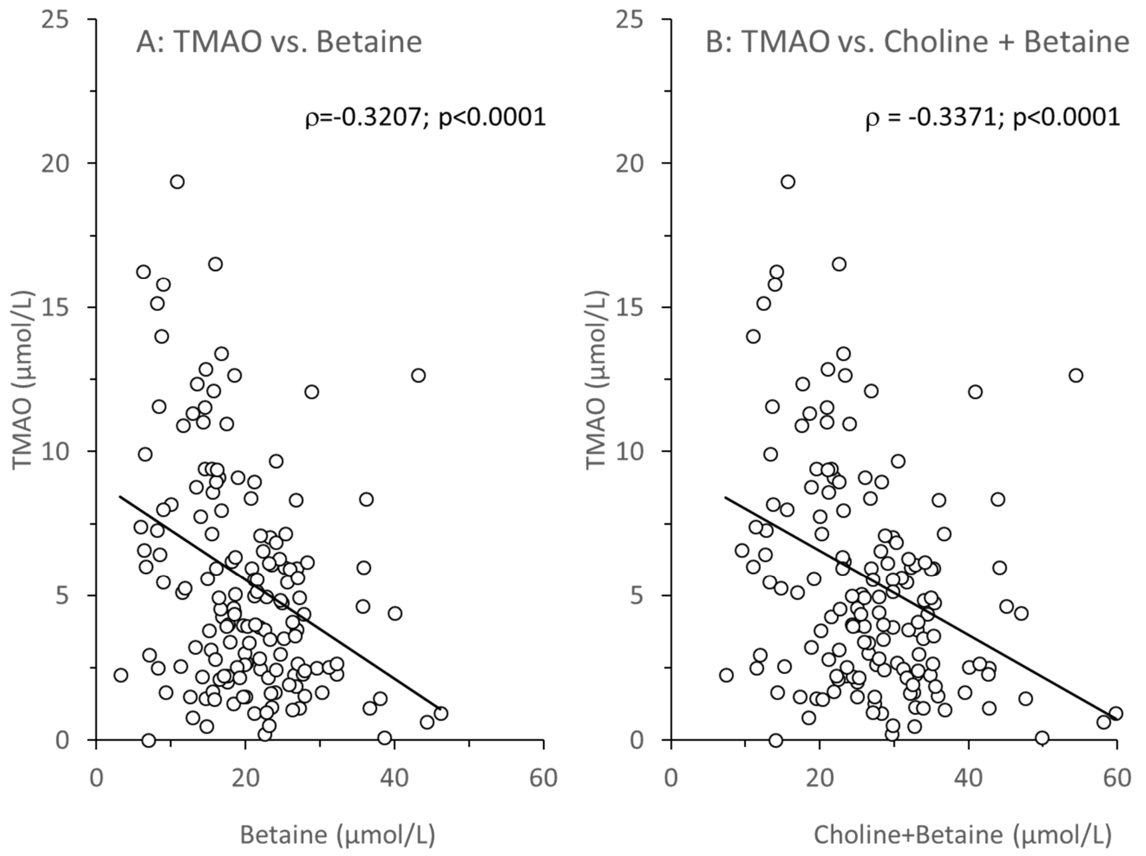

Figure S3: Correlations between TMAO and betaine (A) and the sum of choline and betaine (B) in plasma of CF patients. Data are median values of individual CF patients with (N=146) and without (N=13) exocrine pancreas insufficiency. Abbreviations: ρ, Spearman correlation coefficient; p, significance level.

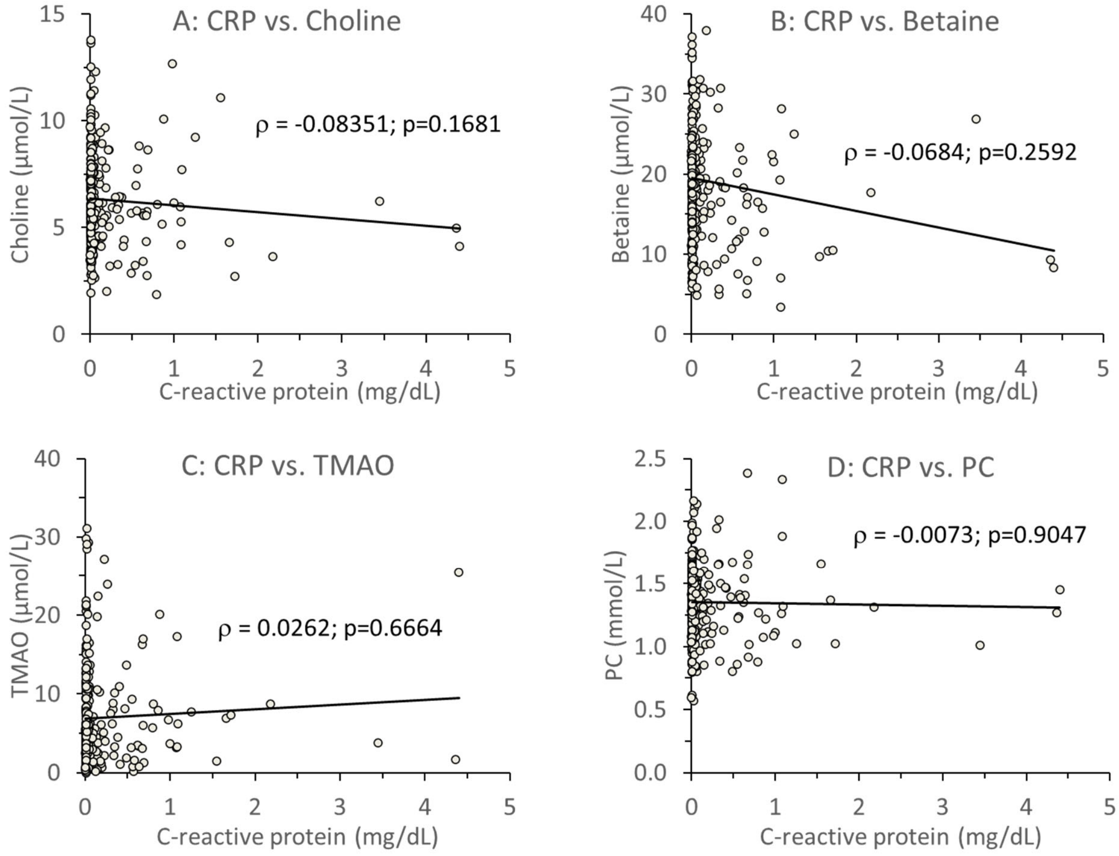

Figure S4: Correlations between C-reactive protein (CRP) and plasma concentrations of choline (A), betaine (B), trimethylamine oxide (TMAO) (C) and phosphatidylcholine (PC) (D) in exocrine pancreas-insufficient (EPI) CF patients. Data are from individual determinations of 274 patient visitations, as CRP was not determined on a routine basis, but only in cases of suspected exacerbated bacterial infection. Abbreviations: ρ, Spearman correlation coefficient; p, significance level.

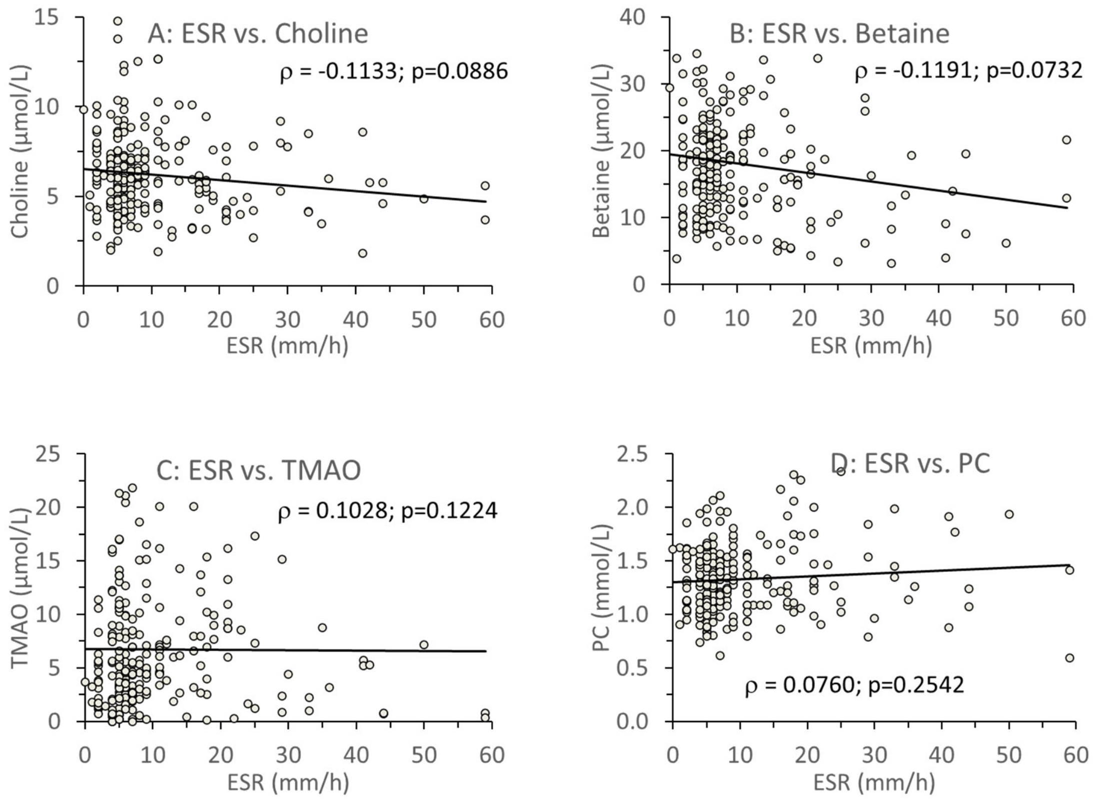

Figure S5: Correlations between erythrocyte sedimentation rate (ESR) and plasma concentrations of choline (A), betaine (B), trimethylamine oxide (TMAO) (C) and phosphatidylcholine (PC) (D) in exocrine pancreas-insufficient (EPI) CF patients. Data are from individual determinations of 227 patient visitations, as ESR was not determined on a routine basis, but only in cases of suspected exacerbated bacterial infection. Abbreviations: ρ, Spearman correlation coefficient; p, significance level.
